# On the Treatment of Puerperal Fever

**Published:** 1831-12

**Authors:** Edwin Long


### PUERPERAL FEVER.

### *On the Treatment of Puerperal Fever.*

By Edwin Long, Esq.

*To the Editor of the London Medical and Physical Journal.*

Sir: Several papers having lately appeared in your valuable Journal upon Puerperal Fever, I cannot resist the temptation of recording the following remedy for that dreadful disease; and, if it prove but as successful in the hands of others as it has now done in mine for twenty years, I shall be amply gratified. I claim not, however, the merit of the discovery; it was the practice of the gentleman whose pupil I was, (then Mr. Thomas Oxley, of this place, now Dr. Oxley, of Bridlington,) and he, I believe, had it from his father, a highly talented and respected practitioner. I do not remember that, during the five years I was with him, we lost a single case, and I know full well that we had several very severe ones; for during part of the time the disease was making dreadful ravages in Leeds.

The remedy in question is in the form of powder, under the head of Pulv. Myrrh. comp., and is composed of equal parts of myrrh, castor, savin, and rue; the dose from three to five grains every three or four hours, according to the severity of the symptoms. I combine it with, as the circumstances of the case may require, some of the class of anodynes, salines, sudorifics, or diuretics, as I may judge expedient. It almost appears to me to exert a specific influence over the uterus, when morbidly excited, as after the peculiar act of parturition; and so great is my confidence in its restorative powers, that I invariably prescribe it, combined with other remedies as before stated, when symptoms occur after delivery which appear to render its employment necessary: these are, pain and tenderness in the hypogastrium; total or partial suppression of the lochial discharge; offensive nature of such discharge; anxious countenance, attended with fever: and I have never, during a pretty extensive practice of twenty years, met with more than two cases where I found it necessary to employ other means.

One of these was certainly a very severe example of the disease: it occurred in 1814, and I then found it necessary to employ both general and local bleeding. So exquisitely tender was the whole abdomen, that the least weight of the bedclothes could not be borne, and it was necessary to raise them by a contrivance made for that purpose. In this case, I remember well, the greatest relief was obtained from spirituous anodyne applications, the cloths being changed every five minutes. The patient, however, ultimately did well.

The only other case where I had recourse to bleeding, I believe, judging from my after experience, would have recovered very well without it; but I remember feeling very anxious, and perhaps not unaccountably so. Let me, however, I pray you, not be misunderstood, or my meaning misconstrued. I adhere pertinaciously to no one line of practice: if I do not clearly see my way, I blush not to call to my assistance the aid of others; for where the life of a fellow creature is at stake, I consider it the bounden duty of every practitioner, as a man and a Christian, so to do.

If I were first called to a patient where all the symptoms ran high, I certainly should not venture to prescribe the remedy above mentioned, without first having recourse to bleeding, either general or local, as might appear necessary, being entirely guided as to the continuance of depletion by the relief afforded. I have invariably found the urgent symptoms very much mitigated, when there has been a return of the lochia, if that discharge has been completely suppressed, or an increase of it, if but partially so.

Two years ago I attended a lady, nineteen years of age, during her first pregnancy. At the expiration of the seventh month, she had the misfortune to receive a blow on one side of her body, from a chair which was accidentally pushed against her. At the moment it gave her excruciating pain, but after a little time it very nearly subsided: no relief was applied for until several days had elapsed, when my assistance was sought. I found a dull uneasy pain, with a slight sense of heat over the part struck: I recommended the application of leeches, the pulse did not warrant general bleeding. The fœtus, which had been very vigorous before the accident, could now scarcely be felt, and in a week or two after the accident labour pains came on. I was sent for in the evening, and was with my patient just three hours, when a child was expelled, which had evidently been dead some days: the funis was completely twisted round its body, and in its expulsion was broken, from its very tender state, at the part where it formed its attachment to the placenta. As there was no hemorrhage, I waited a sufficient time, trying pressure, &c. to induce action of the uterus, but, being disappointed, I was under the necessity of introducing my hand, and removed the placenta as entire as I could; but, from its almost putrescent state, it unavoidably broke down under the action of my fingers: every portion, however, was removed which could be detected. My patient went on remarkably well for the first two days, but on the third the train of untoward symptoms, which have been so often explained, presented themselves; the pain in this case being, as might reasonably be expected, the most severe where the injury had been received. The treatment which has been before detailed was put in practice, and was attended with the happiest results. After the lapse of three or four days, a small portion of membrane, about the size of a crown-piece, was cast off, when every bad symptom disappeared, as if by magic.

I have in several instances witnessed this before, and in one or two cases, where a portion not much larger than a shilling has been expelled, and which I conceive to have acted like an extraneous body, by keeping up irritation. I, therefore, made it an invariable rule to desire the nurse, in cases of this sort, to observe particularly whether any thing of the kind has been discharged, and, if possible, to reserve it for my inspection.

The friends of this lady were particularly anxious about her, in consequence of a severe puerperal case having terminated fatally in the neighbourhood just at the time.

I was lately summoned to a patient, whom I had attended six times before, and who this time particularly dreaded the result: a slight hemorrhage had commenced twenty-four hours before. In about twelve hours she was delivered: there was nothing particularly remarkable in her labour; the placenta was expelled without difficulty, but one side of it was in a very tender state, almost approaching to putrescency. On the second day, the usual symptoms supervened, and were increased by pressure towards the right side, attended with considerable fever and remarkably anxious countenance. The treatment which has been detailed was steadily pursued, with the same happy result.

This patient, as I have before stated, was very fearful that she should not recover from this confinement: she had just lost a relative very similarly affected to herself, and upon whom the depletory system, to its fullest extent, had unfortunately been tried in vain; a state of collapse was induced; reaction never took place, and the patient died from exhaustion.

If I might be allowed to prescribe the mode of treatment, I would say, on the very first commencement of any unfavorable symptoms, begin with the powder, in the dose of from three to five grains every three hours, combined with other remedies which may appear to be indicated; and if an amelioration of the symptoms takes place, it will be all which can be wished; but if, on the contrary, instead of diminishing, they increase in severity, then, of course, if the state of the pulse and other circumstances warrant it, have recourse to the abstraction of blood and other depletory measures. This is the plan I shall, of course, adopt myself, should any such unfortunate case occur in my own practice.

*Pontefract; October* 1831.

---

